# A systematic review of the relationship between magnetic resonance imaging based resting-state and structural networks in the rodent brain

**DOI:** 10.3389/fnins.2023.1194630

**Published:** 2023-07-24

**Authors:** Fatemeh S. N. Mahani, Aref Kalantari, Gereon R. Fink, Mathias Hoehn, Markus Aswendt

**Affiliations:** ^1^Cognitive Neuroscience, Institute of Neuroscience and Medicine (INM-3), Research Centre Juelich, Juelich, Germany; ^2^Department of Neurology, Faculty of Medicine and University Hospital, University of Cologne, Cologne, Germany

**Keywords:** rat, mouse, MRI, DTI, rs-fMRI, graph theory, connectivity

## Abstract

Recent developments in rodent brain imaging have enabled translational characterization of functional and structural connectivity at the whole brain level *in vivo*. Nevertheless, fundamental questions about the link between structural and functional networks remain unsolved. In this review, we systematically searched for experimental studies in rodents investigating both structural and functional network measures, including studies correlating functional connectivity using resting-state functional MRI with diffusion tensor imaging or viral tracing data. We aimed to answer whether functional networks reflect the architecture of the structural connectome, how this reciprocal relationship changes throughout a disease, how structural and functional changes relate to each other, and whether changes follow the same timeline. We present the knowledge derived exclusively from studies that included *in vivo* imaging of functional and structural networks. The limited number of available reports makes it difficult to draw general conclusions besides finding a spatial and temporal decoupling between structural and functional networks during brain disease. Data suggest that when overcoming the currently limited evidence through future studies with combined imaging in various disease models, it will be possible to explore the interaction between both network systems as a disease or recovery biomarker.

## Introduction

### How is the functional and structural network defined and measured?

*Functional connectivity* (FC) is extracted from low-frequency fluctuations (below 0.1 Hz) observed in the blood oxygen level-dependent (BOLD) MRI signal, which serves as a surrogate marker of neuronal activity. During the resting state, i.e., in the absence of an external stimulus, the correlation of the time-series in resting-state functional magnetic resonance imaging (rs-fMRI) data between distinct regions relates to the level of functional connectivity (Biswal et al., 1995). While conventional approaches rely on predefined regions for the connectivity analysis (e.g., seed-based method), independent component analysis (ICA) is used to identify (Jonckers et al., 2011) functional clusters of voxels based on the BOLD signal (Jonckers et al., 2011). There is a temporal delay in neurovascular coupling, which refers to the hemodynamic response, i.e., cerebral rate of oxygen metabolism CMRO^2^ and perfusion (cerebral blood flow and volume), subsequent to neural activation. But compelling mechanistic evidence exists that demonstrates a direct correlation between neural activity and the BOLD signal. This evidence is supported by simultaneous acquisition of electrophysiology data (Thompson et al., 2013) or imaging of calcium dynamics utilizing chemical Ca^2+^-sensors (Pradier et al., 2021) or genetically encoded calcium indicators such as GCaMPs (Ma et al., 2016). However, it should be noted that pathological conditions such as a stroke can perturb blood circulation and neurovascular coupling, thereby directly influencing the BOLD signal (Iadecola, 2004; Sunil et al., 2022).

In spite of variances in animal preparation, anesthesia, and data analysis approaches, a recent multicenter study conducted in rodents has provided compelling evidence that intrinsic connectivity networks (ICNs) spanning the cortex and subcortical regions during anesthesia align closely with those observed in awake humans (Bajic et al., 2017). Specifically, the default mode network (DMN), which plays a prominent role in human brain function, has been found to exhibit analogous patterns in anesthetized rodents (Grandjean et al., 2020). Furthermore, the existence of individual differences in functional connectivity (FC), initially identified in human studies, has been corroborated in awake head-fixed mice, underscoring the potential for subject-specific identification based on the individual variation in the functional network (Bergmann et al., 2020). The integration of complementary techniques such as electrophysiology, and optical and ultrasound imaging fMRI has emerged as an indispensable approach in unraveling the underlying mechanisms of FC in both healthy and pathological conditions (Pais-Roldán et al., 2021; Van der Linden and Hoehn, 2021).

*Structural connectivity* (SC) encompasses the aggregate of axonal and dendritic fibers that establish connections between distinct brain regions. These white matter tracts can be derived from diffusion tensor imaging (DTI), a technique that enables the measurement of water molecule displacement in three-dimensional space. In white matter regions, the diffusion of water molecules exhibits anisotropic behavior, aligning with the highly oriented neuronal architecture, while impeded perpendicularly (Pierpaoli et al., 1996). Through the application of a diffusion tensor model, the DTI data can be utilized to compute various diffusion measures within each voxel, such as fractional anisotropy (FA), mean/radial/axial diffusivity (MD, RD, AD). Subsequently, the reconstructed fiber tracts can be accomplished incrementally, moving from voxel to voxel, by determining streamlines based on local tensor information (referred to as deterministic tractography) or chosen at random from a distribution of possible directions (probabilistic tractography). Deterministic tractography is the most common method for mapping the connectome, while the probabilistic method is better suited for reconstructing and dissecting individual white matter tracts (Sarwar et al., 2019).

It is important to note that tractography is bidirectional and not constrained by synapses, but strongly influenced by the selection of diffusion modeling and related parameters, i.e., step size, cutoff values, maximal number of streamlines (Calabrese et al., 2015; Karatas et al., 2021). Nevertheless, DTI-based fiber tracking was shown in multiple studies to be in line with the spatial organization patterns quantified with the gold standard viral tracing, for selected brain regions such as the hippocampus or sensorimotor cortex (Wu and Zhang, 2016; Pallast et al., 2020; Aswendt et al., 2021), and the whole brain connectome (Calabrese et al., 2015). However, it should be noted that this correlation cannot be generalized and depends on a variety of factors, as shown in a recent comparison. The reconstruction parameters and group-wise network thresholding for example strongly influence the specificity and sensitivity of fiber detection, while the detection of long fiber tracts using DTI remains a general challenge (Sinke et al., 2018).

In this review, we searched for studies in rodents (mice and rats) investigating both structural and functional network measures, including studies investigating the relationship of functional connectivity using resting-state fMRI and studies using DTI or viral tracing data to extract the structural network.

### What is known about the relationship of structure/function from human studies?

In human developmental, healthy, and disease MRI studies, positive correlations have been found for functional and structural connectivity. Structural connections strongly correlate with the presence and strength of functional connections but not vice versa. Interhemispheric FC remains intact or only slightly decreased even in split-brain patients (Uddin, 2013), as well as in rhesus monkeys with a surgical incision of the corpus callosum (cc), and BTBR mice with congenital absence of the cc (O'Reilly et al., 2013; Vega-Pons et al., 2016). Experimental and modeling studies confirmed that the relationship between functional and structural connectivity is complex and that correlations depend on spatial and temporal scales. The majority of human and animal investigations rely on resting-state functional magnetic resonance imaging (rs-fMRI), as it has been shown that the robustness of the association between structural and functional networks is enhanced when low-frequency signals are captured over extended sampling periods (Honey et al., 2010). The correlation is further increased when the linear influence of other regions is removed, i.e., by partial correlations, capturing only “direct” statistical dependencies (Liégeois et al., 2020). In contrast to the “static” property of correlations, further analysis of dynamic functional connectivity, i.e., the temporal variation of FC (strength and variability) have recently been shown to correlate with the related structural network (Liao et al., 2015), although there are limitations in analysis and interpretation (Hutchison et al., 2013).

In contrast to the rodent literature, hundreds of multimodal studies have not only examined the relationship between functional and structural connectivity but have already used the complementary information to guide or validate functional/structural connectivity findings (Zhu et al., 2014). Detailed reviews can be found for example in Jorge et al. (2014), Sui et al. (2014), and Babaeeghazvini et al. (2021). These studies have shown that the coupling of SC and FC is significantly changed during aging and in neuropsychiatric and neurological disorders (Zhu et al., 2014; Wang et al., 2015; Vega-Pons et al., 2016; Damoiseaux, 2017). From a more abstract network perspective, fMRI and DTI have provided clear evidence that the brain resembles a hierarchical system, in which modules (defined by dense short-range connections) are integrated by relatively sparse long-range connections (Meunier et al., 2009; Park and Friston, 2013).

Nevertheless, many open questions about the relationship between functional and structural connectivity remain to be solved. There is no consensus on the best correlation or similarity measure to compare brain-wide FC and SC and the biological interpretation, e.g., of stable FC in the absence of anatomic connections (Wang et al., 2015). There is limited knowledge of the dynamic changes in structural and functional coupling and the related network measures across development, aging, and disease (Scharwächter et al., 2022).

### Current status of rodent structure/function studies

First combined DTI and fMRI measurement in humans and rodents have been reported in the late 1990s (Werring et al., 1998; Silva et al., 1999). The last two decades have seen a rapid development in adapting imaging setups and sequences to perform rs-fMRI and DTI scans in rodents at a comparable quality. As a result, it is now possible to go beyond the boundaries of clinical studies, i.e., to complement MRI with *in vivo* PET, CT, and optical imaging in longitudinal studies (Hoehn and Aswendt, 2013; Pradier et al., 2021; Van der Linden and Hoehn, 2021), and to correlate individual *in vivo* with *ex vivo* data, i.e., gene expression or microscopy (Mills et al., 2018; Goubran et al., 2019). Large-scale explorations of the mouse brain connectome at the cellular and gene expression level have created reference data for hundreds of samples in standardized atlas space using microscopy (Lein et al., 2007), high-resolution anatomical MRI (see summary in Scharwächter et al., 2022), and DTI (Calabrese et al., 2015). By using the gold standard whole-brain viral tracing, the Allen Mouse Brain Connectivity Atlas (Oh et al., 2014), combined with whole-brain rs-fMRI, Stafford et al. (2014) could confirm concordance between structural and functional connectivity in mice as well as the prediction of functional connectivity by the structural foundation.

This review was stimulated by our own multimodal rodent MRI experiments in various disease models (reviewed in Hoehn and Aswendt, 2013; Van der Linden and Hoehn, 2021), for which we found only a minimal number of studies in the literature to compare. Straathof et al. (2019) recently provided a systematic review of the relationship between structural and functional networks across many species, but so far, there is no concise review of the rodent brain connectivity literature.

## Results

### Overview of the systematic review

**Figure 1:**
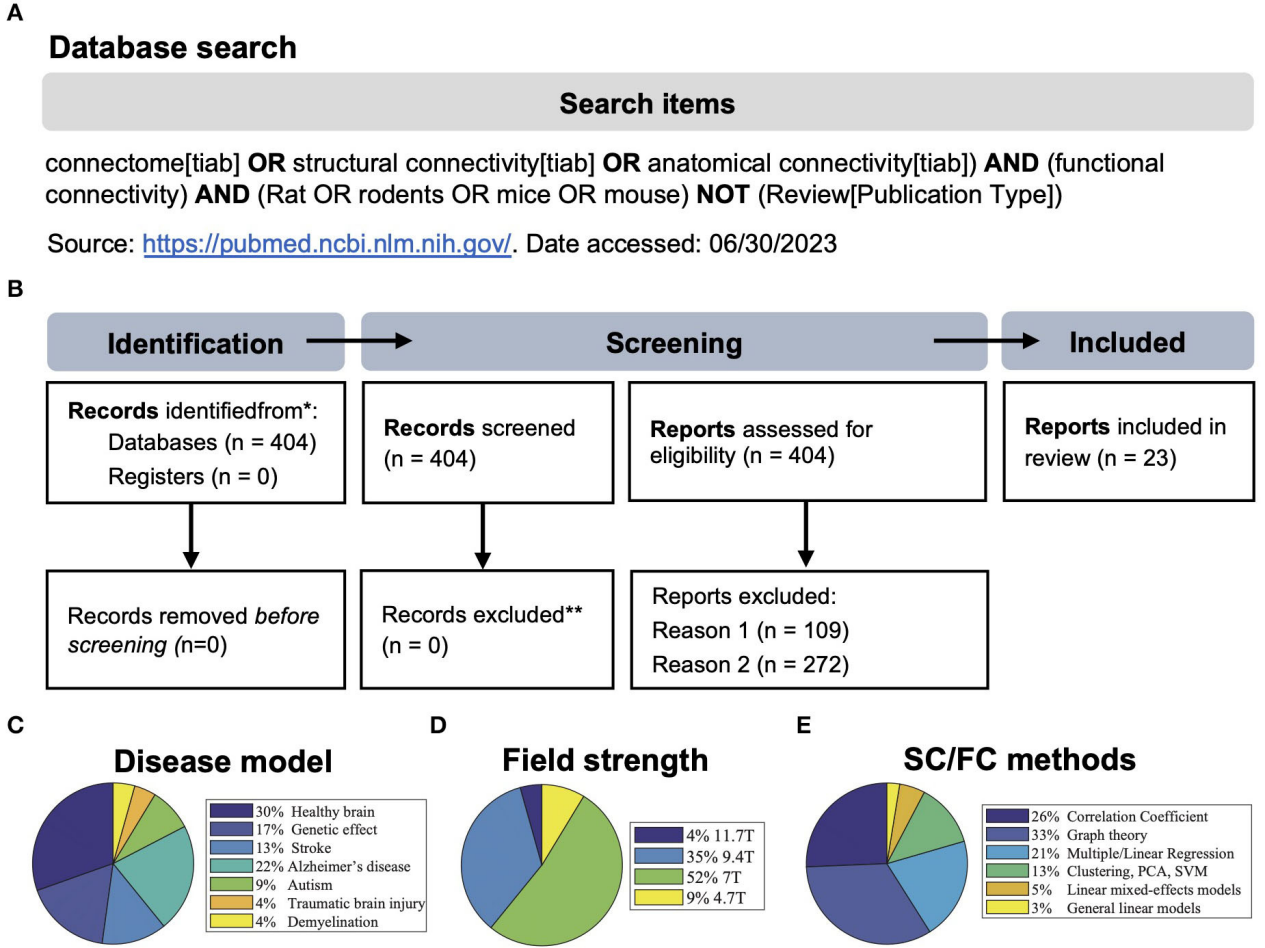
**(A, B)** Search strategy and PRISMA 2020 flow diagram of the systematic review process (modified from Page et al., 2021). Reason 1: studies without MRI, reason 2: studies containing only one of the imaging techniques, or SC and FC were measured separately and without the purpose of multimodal correlation. **(C–E)** Visualization of the frequency of disease models compared to healthy animals, the magnetic field strength used, and the methods used to analyze structural/functional (SC/FC) networks. PCA, principal component analysis; SVM, support vector machine.

We performed a systematic review according to the PRISMA guidelines (Page et al., 2021).^1^ *N* = 404 studies were found according to the search criteria as the main inclusion criteria (Figure 1A). This initial result was manually filtered for pre-set exclusion criteria resulting in *n* = 23 included studies of which *n* = 16 used mice and *n* = 7 used rats (Figure 1B). Exclusion criteria 1: studies containing only one of the imaging methods (*N* = 109). Exclusion criteria 2: SC and FC were measured separately and without the purpose of multimodal correlation (*N* = 272). Despite the relatively strict search criteria, only the minority of studies (*n* = 15) discussed a direct comparison of structural and functional networks in longitudinal studies. If *ex vivo* SC measures, i.e., viral tracing data, were not considered equal to DTI-based fiber tracking, the relevant number would have been even lower (*n* = 10). Most studies (30%) were conducted in healthy rodents, followed by models of Alzheimer's disease (22%) and stroke (13%) and various other neurological or neuropsychiatric disease models and mouse strain comparisons (Figure 1C). Most studies used male rodents and no study compared the influence of sex on the network properties. The mean sample size in studies with mice was 13 ± 3.6 and in studies with rats was 17.1 ± 3.8. The age of mice (excluding *n* = 3 studies related to developmental or aging-related effects) was 3.9 ± 3 months. The age of rats was not reported in all studies but, if available, was 1.9 ± 1.5 months.

In the direct comparison of the material and methods (Table 1, extended version: Supplementary Table 2), we found that despite two studies, all studies were conducted at high field, i.e., ≥7.0 T (Figure 1D), using high-resolution gradient echo (GE) or spin echo (SE) *in vivo* and *ex vivo* sequences for DTI and rs-fMRI ranging from 0.1 to 0.5 mm in-plane image resolution and a slice thickness of 0.3–0.6 mm. There was rather large variability in the number of image volumes acquired for rs-fMRI (150–1,500). Similarly, the b-value, which is relevant for the diffusion weighting, varied between 1,000 and 2,000 s/mm^2^, and the number of diffusion-encoded images (orientations) varied between 15 and 126. Software code/workflows or raw data were only publicly available in *n* = 3 studies. Likewise, the data processing was not described at a level of detail nor shared using version-control tools such as Git, which would allow an independent replication. From the information provided, we speculate that the analysis was performed using in-house developed Python or Matlab scripts based on established tools, such as ANTs, DSI Studio, FSL, and SPM, for data pre-processing, image registration, and connectivity matrix generation (Supplementary Table 3). Across most studies, there was a standard scheme of processing steps, including motion/eddy current correction, spatial smoothing, temporal demeaning, bandpass filtering, and nuisance regression. However, it has to be distinguished again that there were algorithms for which very similar parameters were applied in all studies, e.g., the cutoff at 0.1 Hz for BOLD times series analysis, whereas functional components were either extracted by the individual- or group-wise independent component analysis (ICA) or FC was directly calculated for specific brain regions, derived by co-registration with a standard atlas.

**Table 1:** Summary of experimental groups and imaging protocols in selected studies.

**References**	**Species**	**FS**	**FC**	**SC**	**Disease model**
Arefin et al. (2017)	**Mouse**	7.0 T	*In vivo* MRI	*In vivo* MRI	Psychiatric disorders
Asleh et al. (2020)	**Mouse**	9.4 T	*In vivo* MRI	*In vivo* MRI	Neurofibromatosis type 1
Díaz-Parra et al. (2017)	**Rat**	7.0 T	*In vivo* MRI	*In silico*	None
Degiorgis et al. (2020)	Mouse	7.0 T	*In vivo* MRI	*In vivo* MRI	Alzheimer's disease
Grandjean et al. (2017)	**Mouse**	9.4 T	*In vivo* MRI	*In silico*	None
Green et al. (2018)	**Mouse**	9.4 T	*In vivo* MRI	*In vivo* MRI	Stroke + Stem cells
Green et al. (2019)	**Mouse**	9.4 T	*In vivo* MRI	*In vivo* MRI	Alzheimer's disease
Haberl et al. (2015)	**Mouse**	11.7T	*In vivo* MRI	*In vivo* MRI	Autism
Hübner et al. (2017)	**Mouse**	7.0 T	*In vivo* MRI	*In vivo* MRI	Cuprizone (demyelination)
Karatas et al. (2021)	**Mouse**	7.0 T	*In vivo* MRI	*In vivo* MRI	None
Kesler et al. (2018)	**Mouse**	7.0 / 9.4 T	*In vivo* MRI	Ex vivo MRI	Alzheimer's disease
Mechling et al. (2016)	**Mouse**	7.0 T	*In vivo* MRI	*In vivo* MRI	Opioid receptor knockout
van Meer et al. (2010)	**Rat**	4.7 T	*In vivo* MRI	*In vivo* MRI	Stroke
van Meer et al. (2012)	**Rat**	4.7 T	*In vivo* MRI	*In vivo* MRI	Stroke
Melozzi et al. (2019)	**Mouse**	9.4 T	*In vivo* MRI	*In vivo* MRI	None
Muñoz-Moreno et al. (2018)	**Rat**	7.0 T	*In vivo* MRI	*In vivo* MRI	Alzheimer's disease
Muñoz-Moreno et al. (2020)	**Rat**	7.0 T	*In vivo* MRI	*In vivo* MRI	Alzheimer's disease
Parent et al. (2020)	**Rat**	9.4 T	*In vivo* MRI	*In vivo* MRI	Traumatic brain injury
Schroeter et al. (2017)	**Mouse**	9.4 T	*In vivo* MRI	*In vivo* MRI	None
Sethi et al. (2017)	**Mouse**	7.0 T	*In vivo* MRI	*In silico*	None
Straathof et al. (2020)	**Rat**	9.4 T	*In vivo* MRI	Ex vivo MRI	None
Vega-Pons et al. (2016)	**Mouse**	7.0 T	*In vivo* MRI	*In vivo* MRI	Corpus callosum agenesis
Zerbi et al. (2018)	**Mouse**	7.0 T	*In vivo* MRI	*In vivo* MRI	Autism

BOLD, blood oxygenation level-dependent; CNTNAP2^−/−^, contactin-associated knockout; d, days; EPI, Echo Planar Imaging; Fmr1^−/*y*^, Fragile X Messenger Ribonucleoprotein-1 knockout; FC/SC, functional/structural connectivity; FS, magnetic field strength; m, months; N/A, not applicable; SWS, rat connectome project in Swanson space; SE-DT-EPI, spin-echo diffusion tensor echo planar imaging; wks, weeks; *In silico*, generated from high-resolution brain atlas data.

In summary, the methodological approach primarily related to the generation of functional and structural networks seems to be rather laboratory-specific (Figure 1E). This prevents further unbiased quantitative comparisons of data quality and post-processing strategy.

### Strain- and gene-specific studies of functional and structural connectivity as unrelated networks

Mice and rats are the most frequently used animals in biomedical studies. Their anatomical, physiological, and behavioral differences have been detailed (Ellenbroek and Youn, 2016). By applying a comparable protocol for anesthesia, MR sequences, and analysis, Jonckers et al. (2011) could show that there are differences in the FC between mice and rats, especially when comparing the same number of components using ICA, i.e., the unilateral vs. bilateral cortical components. Furthermore, even for inbred mouse strains (with very high genetic homogeneity), behavioral, neuroanatomical, and brain size differences have been reported consistently (Bothe et al., 2004; Chen et al., 2006; Wahlsten et al., 2006). In this line, the first group of studies (*n* = 8) compared structural and functional networks separately between wild-type and transgenic or knockout mice. Karatas et al. (2021) investigated the functional and structural network differences in two commonly used mouse inbred strains: C57BL/6 and BALB/cJ. Both strains were initially developed in the early 20th century, and are since then commercially kept and distributed as genetically homogeneous inbred strains. In the comparison, Karatas et al. hypothesized that differences between the functional and/or structural networks would explain the known behavioral differences between C57BL/6N and BALB/cJ, and the similarities of the behavior of BALB/cJ mice with certain aspects of autism spectrum disorder. Indeed, structural inter-strain differences were found regarding size and fiber density, e.g., in the corpus callosum, and along cortico-striatal, thalamic, and midbrain pathways. High-resolution fiber mapping (hrFM) showed that the reduced cc fiber density in BALB/cJ was highly variable. However, reduced SC in BALB/cJ did not lead to differences in interhemispheric functional connectivity, which aligns with studies of callosal agenesis, i.e., the absence of direct anatomical connections (Vega-Pons et al., 2016). Strain differences of FC point to other subnetworks than those differences found for SC. Despite many significant differences in FC of selected regions, subnetworks (e.g., DMN), and network organization, there was no direct link to the strain-specific behavioral features.

Zerbi et al. investigated potential developmental differences for two mouse models, the contactin-associated knockout (CNTNAP2^−/−^) and Fragile X Messenger Ribonucleoprotein-1 knockout (Fmr1^−/*y*^) mouse (Zerbi et al., 2018). CNTNAP2 expression starts at E14 and peaks during adulthood, whereas FMR1 is mainly expressed in early embryonic development, which the authors hypothesized to influence selectively the functional and/or SC at the imaging time points P32, P58, and P112. The authors described a similar reduction of FA in the corpus callosum and other major white matter tracts in both genotypes compared to wild type-controls. In CNTNAP2^−/−^ mice at P112, a reduced structural integrity in the cingulum, projecting to the entorhinal cortex, was paralleled by a reduced FC in the limbic network, i.e., also the entorhinal cortex. Unfortunately, the DTI data was not further explored for other brain regions or correlated to the rs-fMRI findings in Fmr1^−/*y*^ mice. However, reduced coupling strength in an extensive symmetric functional network in Fmr1^−/*y*^ mice compared to controls was positively correlated to reduced structural connections between related brain areas using retrograde viral tracing.

In a transgenic rat model of Alzheimer's disease, Muñoz-Moreno et al. (2018) described functional and structural network measures related to cognitive performance. Interestingly, already at an early stage, i.e., before β-amyloid plaque overload, global graph theoretical measures of structural network strength and efficiency were reduced in the transgenic compared to the wild-type control group. In contrast, the functional network (both binarized and weighted) showed no significant differences, despite significantly increased local network measures for selected brain regions, e.g., the right hypothalamus and the ventral tegmental area. The authors reported strong correlations between cognitive performance and brain network measures. However, they did not combine functional and structural information in this comparison.

Even single, targeted deletions have been reported to influence specific functional and structural subnetworks. In knockout mice, lacking the mu opioid receptor (MOR) gene *Oprm1*, functional and, to a much lesser extent, SC in the reward/aversion circuitry (composed of the periaqueductal gray, thalamus, habenula, and nucleus accumbens) was significantly different compared to control mice (Mechling et al., 2016). These *in vivo* results add to the traditional view of MOR mediating the dual analgesic and rewarding effect of morphine toward the importance of pain relief as the primary MOR function. Notably, there was no clear overlap of gene-specific functional and structural network modifications, i.e., higher fiber counts in knockout compared to control mice in the nucleus accumbens, but a loss of its hub function in rs-fMRI analysis. Another example of significant network changes induced by a single gene knockout is a study by Arefin et al. (2017) using mice depleted for the G-protein coupled receptor 88 gene (*Gpr88*), which is linked to neuropsychiatric disorders. Alterations in FC were associated with specific behavioral deficits in the knockout mice, e.g., the suppression of FC in mid-rostral and cortico-subcortical components of the DMN, which could be associated with bipolar disorder or schizophrenia, and increased functional as well as structural caudate putamen to motor cortex connectivity, which could relate to the hyperactivity.

### Structure-function relationship in the healthy brain

This chapter discusses seven publications with a focus on the direct relationship between structural and functional networks in the healthy rodent brain. All studies based their results on resting-state fMRI data. For structural networks, they relied on either diffusion-based MRI, viral tracers, or the neuroanatomic literature. Most studies included whole-brain analysis, while others were limited to the cortex. All studies agree on the general statement of the strong positive relationship between structural and functional networks in the healthy rodent brain. However, the diverse details of each study are worth presenting individually.

**Figure 2:**
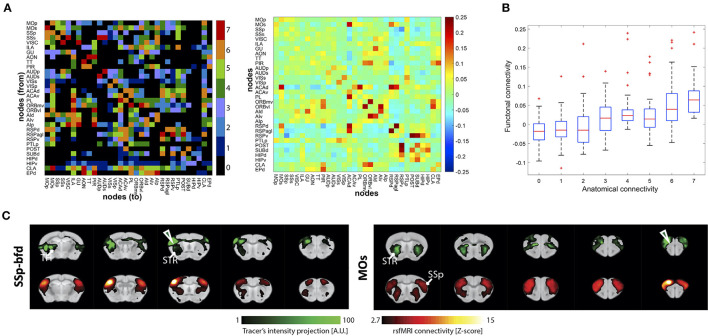
Examples of structural and functional connectivity overlap and mismatch findings from the reviewed studies. **(A)** Structural connectivity (SC) matrix generated from histological tracing data of the rat cortex (left) and functional connectivity (FC) matrix derived from *in vivo* rs-fMRI (right). SC color scale represents the categorical weights of structural links (0, not present; 1, very weak; 2, weak; 3 weak/moderate; 4, moderate; 5, moderate/strong; 6, strong; 7, very strong). FC color scale represents Pearson correlation coefficient of BOLD time course data, which was converted to Fisher's *z*-values. **(B)** Pairwise functional interactions as a function of the underlying SC (“anatomical connectivity”) ranging from 0 to 7 very strong weights of structural links. **(A, B)** was reprinted with permission from Díaz-Parra et al. (2017). **(C)** Qualitative comparison between tracer distribution—reflecting SC (green; top rows) and FC pattern derived from rs-fMRI (red; bottom rows) shown for the injection sites/seeds SSp-bfd () and MOs (). The results illustrate a high degree of similarity between the measurements, particularly in ipsilateral cortico-striatal connectivity. A high degree of overlap was also found in contralateral cortico-cortical connections, whereas cortico-thalamic anatomical projections were not detected by rs-fMRI. **(C)** reprinted under the terms of the CC-BY 4.0 license from Grandjean et al. (2017).

Díaz-Parra et al. (2017) based their rat cortex study on a careful systematic review of the neuroanatomical literature to determine the structural networks. They reported a correspondence between the functional strength and the corresponding structural weight for region connections (Figures 2A, B). They concluded that the strength of the underlying structural connection could predict the functional network intensity between areas. Analysis on the network level showed that densely connected structural motifs directly impact the structure of the functional networks.

The main focus of the study by Kesler et al. (2018) was on a comparison between an Alzheimer's disease mouse model and its healthy litter mates. The 5XFAD transgenic mouse is characterized by amyloid-beta plaque accumulation leading to early neuronal dysfunction and cognitive impairment. The central part of this study deals with the independent comparison of *in vivo* functional (rs-fMRI-based) and postmortem structural (DTI-based) networks between WT and transgenic groups. In a small side-analysis, the authors also studied the direct relationship between structural and functional network data, performing statistical analysis on weighted networks. Based on various graph theoretical variables, a good agreement between both networks was found for the path length. Notably, the correlation within the default mode network was significantly higher in the transgenic AD mice than in the WT littermates.

Straathof et al. (2020) investigated the correlation between functional and structural networks in the healthy rat brain cortex. For structural network data, they used postmortem DTI and neuronal tracer information, based on the NeuroVIISAS database for rat brains. The correlation analysis for interhemispheric connections was significant for both, DTI and neuronal tracers with rs-fMRI data. For intrahemispheric connections, however, the correlation was only significant for DTI data but not for neuronal tracers. Detailed analysis for various subnetworks resulted in a variable agreement between functional and structural (DTI-based) networks.

Schroeter et al. (2017) compared three different mouse strains with varying strengths of interhemispheric corpus callosum (CC) connectivity. I/LnJ mice lack the interhemispheric callosal connection completely, BALB/c mice are known for a tendency for compromised CC integrity, while C57BL/6 mice have a fully developed CC. Based on DTI and rs-fMRI data, the authors reported the loss of interhemispheric FC in I/LnJ mice in correspondence with a lack of CC. For all strains, the cortical FC was correlated with the DTI-based SC for CC.

In the study by Sethi et al. (2017) on the healthy mouse brain, the directed mesoscale mouse connectome from the Allen Brain Connectivity Atlas (based on viral tracers) was applied for the structural network information. The authors primarily focused their analysis on conditions of statistical approaches and data acquisition conditions, e.g., data frequency power, and included computational model calculations. Graph theory-based analysis of degree and clustering coefficient led to the conclusion of a robust correlation between structural and functional networks.

In another study comparing rs-fMRI data with the structural connectome of the Allen Brain Connectivity Atlas of viral tracer data, Grandjean et al. (2017) carefully analyzed individual connections across the healthy whole mouse brain. General good correspondence between structural and functional networks was reported. In a further step, using graph-theoretical approaches, the authors aimed to distinguish monosynaptic and polysynaptic connections of the rs-fMRI data sets, based on the viral tracer structural reference data, demonstrating a higher complexity between structural and functional network systems (Figure 2C). Intrahemispheric structural connections between the isocortex, hippocampus, and striatum were monosynaptic. Interhemispheric homotopic connections of isocortex regions and, to a lesser extent, of hippocampal areas also proved monosynaptic. On the other hand, diverse polysynaptic connections were also found. Subcortical connections emerged as polysynaptic from the analysis. The interhemispheric connections from striatum to isocortex and to contralateral striatum were noted here. Also, functional connections of more spatially extended networks, such as e.g., the DMN, appeared as polysynaptic connections.

In a recent impressive study, Melozzi et al. (2019) used DTI-based structural network data sets to test the relationship with rs-fMRI-based functional connectivity. Moreover, they fed the diffusion-MRI-based structural connectome into The Virtual Mouse Brain (TVMB), an extension of the open-source neuroinformatic platform (The Virtual Brain—TVB) to model a virtual functional connectome. This virtual functional connectome was then compared with the experimental functional network data, derived from the same individual mice. This approach allowed testing whether SC constraints and determines FC. Moreover, the tracer-based Allen Brain connectome was used as a gold standard and compared with the DTI-based SC. The authors found a high predictive power for “the existence of a causal relationship between the structural and the functional connectome” (Melozzi et al., 2019). They noted limitations of the DTI-based connectome when compared to the Allen Brain connectome. In particular, the inclusion of structural features, obtainable only from the viral tracer data, such as fiber directionality, connection strength and patterns, and interhemispheric asymmetry, improved the predictive power for the functional connectome. While most studies were performed on group-averaged data sets, the TVMB modeling was performed on individual data sets showing a highly relevant inter-individual variation in the built FC. In conclusion, the authors claimed that “individual variations define a specific structural fingerprint with a direct impact upon the functional organization of individual brains stressing the importance of using individualized models to understand brain function” (Melozzi et al., 2019).

### Structure-function relationship in the diseased brain

The conclusion from the former chapter was the general consensus of a stable relationship between structural and functional networks in the healthy rodent brain. The present chapter will focus on how this relationship may be affected by brain disease or damage that typically induces structural focal or widespread structural tissue damage. We have analyzed the available literature (total of nine original publications) to find answers to whether the relationship between SC and FC upon transition from healthy to pathophysiological conditions may be altered or even lost and whether damage-induced changes in each network occur in parallel or with differing dynamics.

#### Congenital or acquired loss of white matter tracts

In a pharmacologically-induced model of demyelination using cuprizone in mice, Hübner et al. (2017) described a strong reduction in fiber density along major white matter tracts and voxel-wise decreased FA values during chronic demyelination phase compared to control animals. In the control group, the DMN and TPN have clearly separated clusters within the whole brain FC, with a clear antagonistic relationship between them. The cuprizone-treated mice (CUPR group), presented with an overall lower FC and less prominent DMN and TPN clusters. The CUPR group further showed globally decreased SC and FC relative to control. Considering the structure-function relationship, the authors analyzed the DMN and the sensorimotor network. For the DMN, they found a linear relationship between SC and FC in the control group; this correlation, however, vanished in the CUPR mice. For the sensorimotor network, the effect was quite different: here, a widespread SC change was reported for the CUPR group, while only minor FC changes were observed.

Similar to the Cuprizone-induced corpus callosum demyelination, the mouse strain BTBR is a model of agenesis of corpus callosum (ACC) that leads to the absence of the main bundle of fibers connecting the two hemispheres. This model is described by a complete lack of corpus callosum and severely reduced hippocampal commissure. Vega-Pons et al. (2016) studied this ACC mouse model to determine whether SC or FC is the better discriminator from normal healthy C57BL mice using quantitative *class discrimination with single modality* and *modality comparison* approaches. To this end, they performed a global analysis of structural and functional networks using *in vivo* rs-fMRI and postmortem DTI from BTBR and C57BL animals but without reporting detailed information on the subnetworks. The central finding is that SC is significantly more affected than FC by ACC compared to healthy controls. The authors concluded that the difference between ACC mice and control is more significant for structural than for functional connectivity. In their opinion, this points to a (partial) conservation of the homotopy of the FC despite the severe alterations of the underlying structural networks.

#### Single gene deletion

The subject of Arefin et al. (2017) was the investigation of a single gene alteration or deletion on the whole brain structural and functional networks, analyzed with DTI and rs-fMRI. They selected the mouse model with the deletion encoding GPR88, a G protein-coupled receptor 88 enriched in the striatum. Their global brain data were analyzed focusing on DMN and particularly striato-cortical networks. Structural changes increased fiber density in the striato-cortical pathways, linking striatal and cortical areas. However, no structural loss or reduction of fiber density was observed in the brain. Interestingly, the functional networks presented extensive remodeling of intracortical and cortico-subcortical networks and major DMN alterations. Also, the somatosensory and motor cortical functional networks were affected. Thus, structural and functional connectivities showed opposing changes: the single-gene deletion resulted in structural enhancements restricted to striato-cortical areas but led to widespread functional disturbances.

#### Alzheimer's disease

Here we discuss various transgenic animal models of Alzheimer's disease. However, it must be noted that the different models as well as the choice of animal age set the focus differently on beta amyloid plaques, on tau fibrils, or a mixed co-expression of the two. Thus, focusing on different stages of the overall disease, it may (at least in part) explain the variable findings.

In a small preliminary study of only a few animals, Kesler et al. (2018) compared *in vivo* rs-fMRI and postmortem DTI data of the Alzheimer's disease (AD) model of 5XFAD mice with wild-type littermates. The authors pursued a global analysis using mainly graph theoretical variables. The main findings were higher path lengths in transgenic animals, for both SC and FC. In SC, also lower small-worldness was reported. The correlation analysis between SC and FC showed that SC predicted FC in both groups well, with a more robust correlation coefficient in the Alzheimer's disease models. This may be interpreted that despite alterations in both networks in AD brains relative to healthy brains, the SC/FC relationship was preserved.

Muñoz-Moreno et al. (2018) studied TgF344 transgenic rats, a model of Alzheimer's disease. They recorded *in vivo* DTI and rs-fMRI data at an early age, before significant detection of beta-amyloid plaques, and compared them with wild type littermates. Using global graph theoretical measures, the authors reported different structural organization of the whole brain network in both groups. The structural networks of the transgenic rats showed lower integration and segregation than in control animals, indicating different anatomical connections in AD animals. In contrast, no significant differences were found in the global functional network. This finding shows that structurally different networks can be related to the same or similar functional networks, which can be interpreted as an apparent decoupling between SC and FC in this AD model.

In a recent study by Degiorgis et al. (2020), Thy-Tau22 mice were compared to wild type mice using rs-fMRI and DTI at an early stage of tauopathy at 5 months. This is a time when tau fibrils are already present but the animals do not yet show memory or learning deficits. A general brain-wide increase of functional connectivity was reported; focusing on the hippocampus, amygdala, and isocortical regions, notably the somatosensory cortex, a specific hyperconnectivity was reported for the connecting networks. The structural networks however, showed a decreased fiber density in the white matter channels of the fornix, fimbria, the hippocampus, caudate putamen and the thalamus. These opposite changes of the structural and functional networks in the transgenic animals were interpreted by the authors as due to a compensatory mechanism of the functional network to degenerate structural networks. In some particular subnets, this is alternatively suggested to be a maladaptive mechanism. The authors conclude that the identification of “patterns of brain communication that may be pursued as biomarkers of early tau pathology, before the emergence of memory and learning deficits” (Degiorgis et al., 2020).

Green et al. (2019) investigated the effect of elevated tau-protein on SC and FC using a mouse model of tauopathy with regulatable soluble and aggregated human tau-protein. Mice were investigated with DTI and rs-fMRI at 12 months of age when the human tau expression had elevated tau-protein levels in either soluble or aggregated forms in two different transgenic mice, so-called anti-aggregant and pro-aggregant strains. Following, doxycycline was applied for 8 weeks to completely switch off the mutant tau-protein so that soluble tau and most aggregates had disappeared. At that time, SC and FC were re-recorded. Whole brain correlation maps were generated for both networks at both experimental time points of the two tauopathy model groups and corresponding normal littermates. This proper longitudinal study allowed to assess intraindividual SC and FC changes between different (patho-) physiological conditions during disease and treatment and to compare them with healthy littermates of the same age. The FC strength was substantially reduced in the pro- and anti-aggregant animals at full expression of the elevated tau-protein levels, relative to the control group. After doxycycline treatment, when human soluble or aggregated tau had disappeared, FC strength recovered and became indistinguishable from the control group.

In contrast, the structural networks showed only minor, i.e., non-significant, differences from the control group before and after doxycycline treatment. This finding demonstrates that high tau-protein elevation and aggregation have a massive impact on FC without significantly affecting SC, thus pointing again, similar to the study by Muñoz-Moreno et al. (2018) to a disease-induced decoupling of SC and FC. Interestingly, the effective dissolution of human tau resulted in a normalization of FC while the SC remained widely stable. Thus, the SC-FC relation, as existent in healthy normal brains, was recovered by the treatment.

#### Stroke

Two publications from the Dijkhuizen lab (van Meer et al., 2010, 2012) studied functional network changes after stroke in the rat and combined them with data about structural tissue changes. In the longitudinal study (van Meer et al., 2012), recording rs-fMRI and DTI in rats between 1 and 10 weeks post-stroke induction, DTI was only used to calculate fractional anisotropy (FA). Thus, only indirect information about structural tissue alterations was reported, without structural network information. The second publication (van Meer et al., 2010) combined rs-fMRI with manganese-enhanced MRI (MEMRI) in rats in the chronic phase at 10 weeks after stroke induction. MEMRI has been established as an MR imaging modality with manganese as a surrogate marker of Ca2+ influx, i.e., neural activity, and axonal tracer generating a sensitive but low-resolution MRI contrast (Osanai et al., 2022). Van Meer analyzed the intracortical and intercortical FC and studied the MEMRI signal dissipation from the contralateral cortex using it as a structural network proxy. In large cortico-striatal lesions, a functional hyperconnectivity in the healthy hemisphere and a functional hypoconnectivity in the ischemic hemisphere were noted. For small ischemic lesions, where the sensorimotor cortex remained outside the primary lesion core, a hyperconnectivity was found in the ischemic hemisphere's cortex. Structural changes observed by MEMRI were reflected by enhanced signals in the healthy cortex, in agreement with functional hyperconnectivity. The interhemispheric structural MEMRI data showed a reduced transcallosal signal in line with reduced FC between the contralateral M1 and ipsilateral sensorimotor cortex. The authors concluded that FC is closely associated with structural connectivity.

In a longitudinal study, Green et al. (2018) monitored DTI and rs-fMRI repetitively over 12 weeks post-stroke induction, starting from the pre-stroke healthy condition in the same animals. In a second group, stem cell implantation 1 week after stroke was added. The authors determined functional correlation coefficient matrices and fiber density matrices, focusing on sensorimotor networks. A sharp decrease of FC extending across both hemispheres was seen within the first week and persisted for the whole 12 weeks of observation. In mice with stem cell implantation, functional networks were stabilized directly after implantation as long as the cell vitality was observed, pointing to a paracrine effect as an early supportive mechanism of the graft. SC analysis showed fiber-density increases developing within the first weeks after stroke between the cortex and white matter regions, occurring predominantly on the ischemic hemisphere.

Further, a delayed increase of fiber density between the motor cortex and white matter regions, the peduncle, the fornix, and the anterior commissure was seen in the contralateral hemisphere only during the third month. The fiber-density changes were nearly identical for both study groups, independent of stem cell-induced FC stabilization. Thus, the same SC but fundamentally different FC between both groups underscores the need to collect information on structural and functional networks, as both networks may well be decoupled from each other in their response to a pathophysiological event. These findings led the authors to conclude “that under the condition of a stroke, there is an apparent decoupling between structural and functional networks: SC damage does not provide a straightforward explanation for findings of weakened or even defective functional connectivity” (Green et al., 2018).

Interestingly, the decoupling process between both networks is dynamic: direct and persistent FC changes were followed by SC changes only in the first weeks and a second wave after many weeks. Moreover, these structural changes were only very selective, whereas FC changes were seen globally. In the stem cell-treated group, FC stabilization was accompanied by still the same delayed increase in fiber density.

## Discussion

This review confirms the existence of robust distinct structure-function relationships in the healthy rodent brain (Straathof et al., 2020). Distinctions under which detailed analysis conditions this statement holds must still be considered in finer future analyses. Due to the strict literature search terminus, we cannot exclude that other rodent SC-FC studies were overseen. Furthermore, in recognition of the limitations inherent in relying solely on keyword-based searches, it is important to acknowledge that our study's search strategy may not have captured all relevant papers on the topic. Human studies, which have been discussed in detail elsewhere (Park and Friston, 2013; Fukushima et al., 2018; Wein et al., 2021) were outside the focus of the present review.

**Figure 3:**
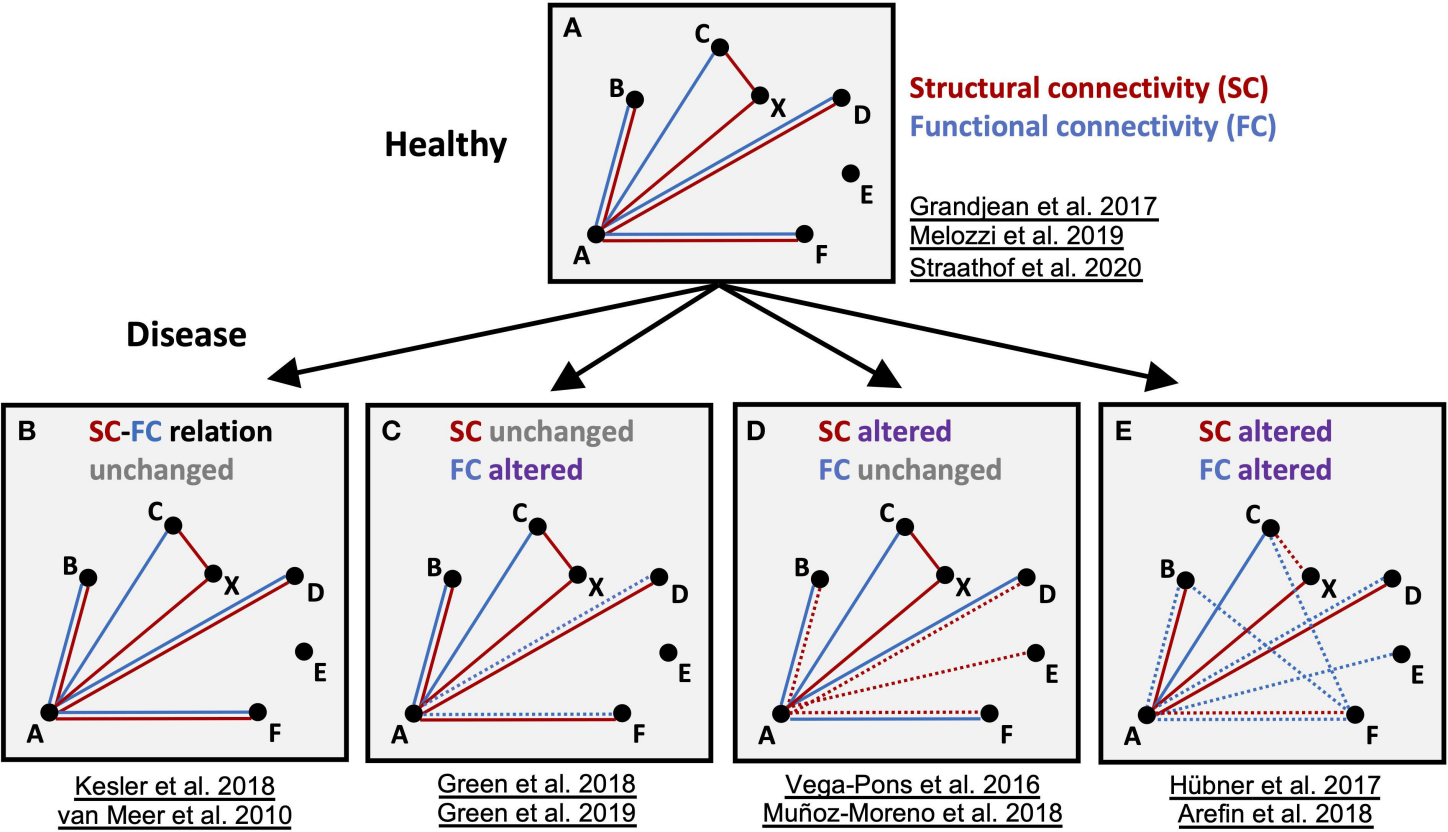
Schematic summary of the findings for structural and functional networks in healthy and disease models. Schematic graph of nodes **(A–E)** and edges connecting the nodes, representing functional (FC, blue) and structural (SC, red) connectivity, respectively. Altered connections are highlighted as dashed lines. In the diseased brain, either fc and/or sc in specific nodes are altered. Importantly, depending on the type of disease and time point, all variants of sc-fc changes can appear and disturb sc-fc coupling present in the healthy state. Represented studies for the four categories are listed.

The presently drawn conclusions, extracted from the individual original publications, summarize the findings concerning the transitions of the structural and functional networks between healthy and diseased brains (Figure 3). Despite varying analyses, strategies and limitations to sometimes selective subnetworks (e.g., DMN or sensorimotor network), the rather stable relationship between healthy SC and FC was found to be disturbed or even lost in the pathophysiological situations. Some authors explicitly described this observation as an apparent decoupling between SC and FC in the condition of diseased brain. Except for one original publication (Green et al., 2018), no longitudinal study examined more than one pathophysiological time point which would, however, be essential to unravel the dynamic of any SC/FC decoupling during the pathophysiological cascade of events during a disease development (Figure 3). Taken together, present knowledge and understanding of pathology-induced alterations of SC and FC networks suggest that they supply complementary network information.

### Methodological limitations

Optimization of imaging protocols is critical to reliably identifying anatomically-aligned connections. For this, recent advances in small animal MRI hardware, including stronger gradients and more sensitive coils, e.g., cryogenic coils with an approximate 2–3-fold SNR increase, have been crucial (Hoehn and Aswendt, 2013). Whereas human MRI protocols have addressed and partially standardized the tradeoffs of scanning time, image resolution, signal specificity, reliability, and harmonized post-processing (Wang et al., 2011; Soares et al., 2013; Smitha et al., 2017; Cao et al., 2019; Haddad et al., 2019), similar standardization efforts for small animal MRI are lacking behind (Mannheim et al., 2018). Firstly, clinical protocols cannot be translated directly to the rodent brain, as up to a 2,800-fold difference in size must be compensated (Hikishima et al., 2017). Secondly, macroscopic differences in anatomy need to be considered. For example, the mouse brain has a gray-to-white matter ratio of 90:10, whereas it is 40:60 in the human brain (Krafft et al., 2012). As lipids in the white matter are dominantly responsible for the diffusion signal (Leuze et al., 2017), rodent diffusion protocols must be many times more sensitive. There is an ongoing discussion on the best approach for high-resolution fiber tracking in rodents, presented here by the large variety of protocols in the reviewed studies. For example, the number of diffusion directions was commonly set to 30, previously described as a good compromise between image quality and scanning time for human DTI based on a Monte Carlo simulation study (Jones, 2004; Soares et al., 2013). However, a different simulation study optimized for mouse brain DTI (Anderson et al., 2020) found that 60 directions acquired with 0.043 or 0.086 mm spatial resolution approximate well the reference connectome acquired with 120 angular samples at 0.043 mm spatial resolution (Calabrese et al., 2015).

Similar requirements in terms of spatial resolution apply to rs-fMRI in rodents. Besides, in rs-fMRI, the imaging protocol determines spatial sensitivity. Whereas GE-EPI is primarily sensitive to large veins, thus spatially less specific, SE-EPI is more sensitive to small vessels, leading to higher spatial sensitivity. The tradeoff with SE-EPI is a lower BOLD CNR and longer acquisition times (Mandino et al., 2019).

Even more critical in rodent studies is maintaining stable physiology, for which most studies rely on anesthesia, which can affect neurovascular coupling in different ways (Pan et al., 2015). The currently most widely used and stable anesthesia protocol (Grandjean et al., 2020), which resembles well-functional networks obtained in awake rodents (Paasonen et al., 2018), is a combination of 0.05 mg/kg bolus/0.1 mg/kg/h i.v. infusion of Medetomidine and 0.5% Isoflurane (Grandjean et al., 2014). As we could show in rats at 11.7 T, motion remains the primary noise source and requires careful regression, e.g., through the simultaneously acquired respiration rate (Kalthoff et al., 2011), or signal regression from ventricular and vascular masks (Grandjean et al., 2020). More recent protocols for awake rs-fMRI might overcome the additional effects of anesthesia; however, they require extensive training of the rodent to accommodate head fixation but do not overcome motion noise (Tsurugizawa et al., 2020).

Despite common challenges in harmonizing imaging setups and protocols, in which rodent MRI is no exception (Mannheim et al., 2018; Gozzi and Zerbi, 2023; Tavares et al., 2023), a recent multicenter study proved the detectability of stable, functional networks in 17 mice rs-fMRI datasets using a standard image processing and analysis pipeline (Grandjean et al., 2020). A similar DTI fiber tracking study is pending; however, there are promising first attempts to improve reproducibility for the specific needs of DTI in small animals (Jelescu et al., 2022), as it has been done for clinical protocols (Grech-Sollars et al., 2015). Besides the agreement and adoption of quality standards for acquisition (Tavares et al., 2023), we expect a large variability in the results of the selected studies to be related to the differences in post-processing and application of more advanced analysis methods, i.e., graph theory (Scharwächter et al., 2022). The priority should be to adopt best practices in data analysis as initially described for human neuroimaging and to make the imaging data available (Nichols et al., 2017). Here, only two out of 22 studies shared the data in an online repository, which is unfortunately true for most rodent MRI studies and strictly limits a more detailed meta-analysis (Mandino et al., 2019).

## Outlook

In conclusion, this review confirmed prior evidence of typical features of SC-FC correlation in the healthy brain (Straathof et al., 2019). The correlation in the diseased brain is, however, far more complex and seems to uncouple under specific conditions, i.e., the existence of interhemispheric FC despite strongly reduced SC (Vega-Pons et al., 2016; Karatas et al., 2021). From the presently available reports on different brain diseases, general pattern and intensity of SC-FC relation changes do not emerge, suggesting that the changes are disease-specific. In future larger, multimodal rodent studies, it will be important to identify the cellular correlate of these changes, e.g., by combining information from viral tracing and 3D histology (Goubran et al., 2019). The presently most detailed analysis was found here in the study by Grandjean et al. (2017), pointing out the particular situations for mono- and polysynaptic connections. In general, future complete analysis will require the investigation of the SC/FC relationship, probing stepwise individual connections across the whole brain and answering varying levels of the robustness of the SC/FC relationship within individual subnets, as already reported in Straathof et al. (2020).

In particular, SC-FC coupling should be investigated at all levels, which is only possible in longitudinal *in vivo* imaging studies. As suggested by Green et al. (2018), the dynamic process evolving over time should not be neglected, indirectly pointing to the possibility that the intensity of SC-FC decoupling may vary with time as the disease progresses. If confirmed in future extensive translational studies, this could indeed be used to predict disease progression and develop individualized network-informed therapeutic strategies.

## Data availability statement

The original contributions presented in the study are included in the article/Supplementary material, further inquiries can be directed to the corresponding author.

## Author contributions

FM, AK, MH, MA, and GF contributed to the conception and design of the study. FM, MH, and MA performed the systematic review. FM, AK, and MA analyzed and summarized the post-processing and analysis methods. MA, MH, AK, and FM wrote the first draft of the manuscript. MA, MH, and GF wrote the final version of the manuscript. All authors contributed to the manuscript revision, read, and approved the submitted version.

## Footnotes

^1^PRISMA guideline checklist provided as Supplementary Table 1.
