# Supplementary material for: A systematic review of the relationship between magnetic resonance imaging based resting-state and structural networks in the rodent brain
Source: Front Neurosci. 2023 Jul 24;17:1194630. doi: 10.3389/fnins.2023.1194630 (PMC10405456; doi:10.3389/fnins.2023.1194630)
Supplement: Supplementary file 1 [file Data_Sheet_1.docx]

# Supplementary Material

**Table 1: PRISMA 2020 for Abstracts checklist^1^**

| Section and topic | Item # | Checklist item | *Notes^2^* |
| --- | --- | --- | --- |
| **Title** |  |  |  |
| Title | 1 | Identify the report as a systematic review. | *Title* |
| **Background** |  |  |  |
| Objectives | 2 | Provide an explicit statement of the main objective(s) or question(s) the review addresses. | *Provided in introduction* |
| **Methods** |  |  |  |
| Eligibility criteria | 3 | Specify the inclusion and exclusion criteria for the review. | *Provided in results section* |
| Information sources | 4 | Specify the information sources (e.g., databases, registers) used to identify studies and the date when each was last searched. | *Provided in results section* |
| Risk of bias | 5 | Specify the methods used to assess risk of bias in the included studies. | *N/A* |
| Synthesis of results | 6 | Specify the methods used to present and synthesize results. | *Descriptive statistics* |
| **Results** |  |  |  |
| Included studies | 7 | Give the total number of included studies and participants and summarize relevant characteristics of studies. | *Provided in results section (n=263)* |
| Synthesis of results | 8 | Present results for main outcomes, preferably indicating the number of included studies and participants for each. If meta-analysis was done, report the summary estimate and confidence/credible interval. If comparing groups, indicate the direction of the effect (i.e., which group is favoured). | *Primary outcomes presented, including the sample number per group in results section. No meta-analysis.* |
| **Discussion** |  |  |  |
| Limitations of evidence | 9 | Provide a brief summary of the limitations of the evidence included in the review (e.g., study risk of bias, inconsistency and imprecision). | *N/A* |
| Interpretation | 10 | Provide a general interpretation of the results and important implications. | *Included in results.* |
| **Other** |  |  |  |
| Funding | 11 | Specify the primary source of funding for the review. | *Included in acknowledgements* |
| Registration | 12 | Provide the register name and registration number. | *N/A* |

^1^ This abstract checklist retains the same items as those included in the PRISMA for Abstracts statement published in 2013,54 but has been revised to make the wording consistent with the PRISMA 2020 statement and includes a new item recommending authors specify the methods used to present and synthesize results (item #6). The checklist was downloaded from<http://www.prisma-statement.org/Extensions/Abstracts>

^2^ Notes reflect additional information provided by the authors. N/A = not applicable.

**Table 2: Summary of experimental groups and imaging protocols in selected studies.**

|  | **Study (Refer-**  **ence)** | **Species (age/sex/ number per group)** | **Scan-**  **ner** | **Functional connectivity protocol** | **Structural connectivity protocol** | **Disease model** | **Code / Data available** |
| --- | --- | --- | --- | --- | --- | --- | --- |
| 1 | [(Arefin et al., 2017)](https://paperpile.com/c/X6NvQ0/PeFtp) | **Mouse**  Gpr88^-/-^  7–8 wks / ♂ n=14 | 7.0 T Bruker Biospec | ***In vivo***  GE-EPI, 0.15 x 0.15 x 0.7 mm, 200 rep. | ***In vivo***  HARDI, 4-shot DT-EPI*,* 0.094x 0.094x0.5 mm, 30 directions, b-factor= 1000 s/mm^2^ | Neuropsychiatric disorders-related | No1 / No |
| 2 | [(Asleh et al., 2020)](https://paperpile.com/c/X6NvQ0/6fV2V) | **Mouse**  Plp-Nf1^fl/+^, C57BL/6  8wks / NA / n=15, 14 | 9.4 T Bruker Biospec | ***In vivo***  SE-EPI, 0.15 x 0.15 x 0.45 mm, 200 rep. | ***In vivo***  SE-EPI*,* 0.1x0.1x0.4 mm, 30 directions, b-factor=1000 s/mm^2^ | Neurofibromatosis type 1 | No / [Yes](https://openneuro.org/datasets/ds003027) |
| 3 | [(Degiorgis et al., 2020)](https://paperpile.com/c/X6NvQ0/MVuK) | Mouse  Thy-Tau22, littermate WT control  5 m / ♂ n=12, 9 | 7.0 T Bruker Biospec | In vivo  GE-EPI, 0.14 x 0.22 x 0.4 mm, NA rep. | In vivo  HARDI, 4-shot DT-EPI, 0.14 x 0.22 x 0.5 mm, 30 directions, b-factor= 500-2000 s/mm2 | Alzheimer’s disease | No / No |
| 4 | [(Díaz-Parra et al., 2017)](https://paperpile.com/c/X6NvQ0/zDEWA) | **Rat**  Wistar NA / NA / n=14 | 7.0 T Bruker Biospec | ***In vivo***  GE-EPI 0.5 mm isotropic, 300 rep. | ***In silico***  TVB simulations with SwS structural atlas as input | None | No / No |
| 5 | [(Grandjean et al., 2017)](https://paperpile.com/c/X6NvQ0/asR0z) | **Mouse**  C57BL/6  NA / ♀ / n=14 | 9.4 T Bruker Biospec | ***In vivo***  GE-EPI, 0.3 x 0.3 x 0.3 mm, 360 rep. | ***In silico***  Allen Mouse Brain Connectivity Atlas | None | [Yes](http://doi.org/10.5905/ethz-1007-59) / Yes |
| 6 | [(Green et al., 2018)](https://paperpile.com/c/X6NvQ0/Ynryp) | **Mouse**  NMRI  12–14 wks / ♂ / n=15 | 9.4 T Bruker Biospec | ***In vivo***  GE-EPI, 0.18 x 0.18 x 0.5 mm, 150 rep. | ***In vivo***  Q-Ball SE-EPI*,* 0.14x0.14x0.5 mm, 126 directions, b-factor=2000 s/mm^2^ | Stroke+Stem cell implantation | No / No |
| 7 | [(Green et al., 2019)](https://paperpile.com/c/X6NvQ0/YRk9u) | **Mouse**  TauRD  10–12 m / ♀ / n=21 | 9.4 T Bruker Biospec | ***In vivo***  GE-EPI, 0.18 x 0.18 x 0.5 mm, 150 rep. | ***In vivo***  Q-Ball SE-EPI*,* 0.14x0.14x0.5 mm, 126 directions, b-factor=2000 s/mm^2^ | Alzheimer’s disease | No / No |
| 8 | [(Haberl et al., 2015)](https://paperpile.com/c/X6NvQ0/dv9D2) | **Mouse**  Fmr1^−/y^ & wild-type  9-12 wks / ♂ / n = 7, 10 | 11.7T Bruker Biospec | ***In vivo***  SE-EPI, 0.26 x 0.26 x 0.5 mm, 600 rep. | ***In vivo***  4-shot SE-EPI, 0.156x0.156x0.5 mm, 30 directions, b-factor=1000 s/mm^2^ | Autism | No / No |
| 9 | [(Hübner et al., 2017)](https://paperpile.com/c/X6NvQ0/KoIop) | **Mouse**  C57BL/6N  7 wks / ♀ / n=16 | 7.0 T Bruker Biospec | ***In vivo***  GE-EPI 0.15 x0.15 x 0.7 mm isotropic, 200 rep. | ***In vivo***  4-shot DT-EPI, 0.1x0.1x0.5 mm, 30 directions, b-factor=1000 s/mm^2^ | Cuprizone (demyelination) | [DTI](http://https//www.uniklinik-freiburg.de/mr-en/research-groups/diffperf/fibertools.html) / No |
| 10 | [(Karatas et al., 2021)](https://paperpile.com/c/X6NvQ0/V3eCI) | **Mouse**  C57BL/6N, BALB/cJ  8-9 wks / ♀ / n=11, 14 | 7.0 T Bruker Biospec | ***In vivo***  GE-EPI 0.15 x0.15 x 0.7 mm isotropic, 200 rep. | ***In vivo***  4-shot DT-EPI, 0.1x0.1x0.5 mm, 30 directions, b-factor=1000 s/mm^2^ | None | No / No |
| 11 | [(Kesler et al., 2018)](https://paperpile.com/c/X6NvQ0/FSXTY) | **Mouse**  5XFAD, C57Bl/6 and SJL background  23 wks / ♂ / n=8 | 7.0 T / 9.4 T Bruker Biospec | ***In vivo***  GE-EPI, 0.25 x 0.25 x 0.5 mm, 450 rep. | ***Ex vivo***  SE, 0.1 mm isotropic, 20 directions, b-value=1000 s/mm^2^ | Alzheimer’s disease | No / No |
| 12 | [(Mechling et al., 2016)](https://paperpile.com/c/X6NvQ0/xCbUX) | **Mouse**  Oprm1^-/-^and wildtype control 12 wks / ♂ / n=16 | 7.0 T Bruker Biospec | ***In vivo***  GE-EPI, 0.15 x 0.15 x 0.7 mm | ***In vivo***  HARDI, 4-shot DT-EPI*,* 0.94x0.94x0.5 mm, 30 directions, b-factor=1000 s/mm^2^ | Mu opioid receptor knockout | No / No |
| 13 | [(van Meer et al., 2010)](https://paperpile.com/c/X6NvQ0/kIUdG) | **Rat**  Spraque Dawley  NA / ♂ / n=18 | 4.7 T Varian | ***In vivo***  GE-EPI, 0.5 mm isotropic | ***In vivo***  T1-weighted MRI after injection of MnCl_2_ | Stroke | No / No |
| 14 | [(van Meer et al., 2012)](https://paperpile.com/c/X6NvQ0/ikjNG) | **Rat**  Spraque Dawley  NA / ♂ / n=24 | 4.7 T Varian | ***In vivo***  GE-EPI, 0.5 x 0.5 x 1.5 mm, 1200 rep. | ***In vivo***  SE-DT-EPI, 0.5 mm isotropic, 50 directions, b-factor=1250 s/mm^2^ | Stroke | No / No |
| 15 | [(Melozzi et al., 2019)](https://paperpile.com/c/X6NvQ0/Qcny4) | **Mouse**  B6129PF/J1  NA / ♂ / n=19 | 9.4 T Bruker Biospec | ***In vivo***  SE-EPI, 0.15 x 0.15 x 0.45 mm | ***In vivo***  SE-DT-EPI, 0.1x0.1x0.4 mm, 30 directions, b-factor=1000 s/mm^2^ | None | [Yes](https://openneuro.org/datasets/ds002307) / No |
| 16 | [(Muñoz-Moreno et al., 2018)](https://paperpile.com/c/X6NvQ0/zmTU3) | **Rat**  TgF344-AD and Fischer  NA / ♂ / n=18 | 7.0 T Bruker Biospec | ***In vivo***  GE-EPI, 0.4 x 0.4 x 0.6 mm, 600 rep. | ***In vivo***  SE-EPI, 0.31x0.31x0.31 mm, 60 directions, b-factor=1000 s/mm^2^ | Alzheimer’s disease | No / No |
| 17 | [(Muñoz-Moreno et al., 2020)](https://paperpile.com/c/X6NvQ0/mNt8B) | **Rat**  TgF344-AD and Fischer  NA /♂ / n=18 | 7.0 T Bruker Biospec | ***In vivo***  GE-EPI, 0.4 x 0.4 x 0.6 mm, 600 rep. | ***In vivo***  SE-EPI, 0.31 isotropic, 60 directions, b-factor=1000 s/mm^2^ | Alzheimer’s disease | No / No |
| 18 | [(Parent et al., 2020)](https://paperpile.com/c/X6NvQ0/T4ISq) | **Rat**  Spraque Dawley  23–24 d / ♂ / n=16 | 9.4 T Bruker Biospec | ***In vivo***  GE-EPI, 0.5 x 0.5 x 0.5 mm, 300 rep. | ***In vivo***  EPI, 0.5 x 0.5 x 0.5 mm, 15 directions, b-factor=1000 s/mm^2^ | Traumatic brain injury | No / No |
| 19 | [(Schroeter et al., 2017)](https://paperpile.com/c/X6NvQ0/aOp2I) | **Mouse**  C57BL/6 and BALB/c / 3–4 m / ♀ / n=10,9 | 9.4 T Bruker Biospec | ***In vivo***  GE-EPI, 0.2 x 0.2 x 0.5 mm, 360 rep. | ***In vivo***  multi-shot DT-EPI, 0.26x0.23x0.5 mm, 36 directions, b-factor=1000 s/mm^2^ | None | No / No |
| 20 | [(Sethi et al., 2017)](https://paperpile.com/c/X6NvQ0/gY8nt) | **Mouse**  C57BL/6J  2 m / ♂ / n=18 | 7.0 T Bruker Biospec | ***In vivo***  GE-EPI, 0.22 x 0.2 x 0.4 mm, 360 rep. | ***In silico***  Mesoscale connectome derived from Allen Mouse Brain Connectivity Atlas | None | No / No |
| 21 | [(Straathof et al., 2020)](https://paperpile.com/c/X6NvQ0/ihMYD) | **Rat**  Wistar  12 wks / ♂ / n=12 | 9.4 T Varian MR system | ***In vivo***  GE-EPI, 0.6 mm isotropic, 800 rep. | ***Ex vivo***  HARDI 8-shot 3D EPI, 0.15 mm isotropic, 60 directions, b-value=3842 s/mm^2^ | None | No / No |
| 22 | [(Vega-Pons et al., 2016)](https://paperpile.com/c/X6NvQ0/kRcQO) | **Mouse**  BTBR and C57BL/6  26 wks /♂ / n=10, 10 | 7.0 T Bruker Pharmascan | ***In vivo***  EPI, 0.23 x 0.23x0.75 mm, 360 rep. | ***In vivo***  GE-EPI, 0.13x0.13x0.35 mm, 81 directions, b-factor=1262 s/mm^2^ | agenesis of the corpus callosum | No / No |
| 23 | [(Zerbi et al., 2018)](https://paperpile.com/c/X6NvQ0/Yd1y4) | **Mouse**  Fmr1^−/y^ and CNTNAP2^−/−^  34-112 d / ♂ ♀ / n=13, 14 | 7.0 T Bruker Pharmascan | ***In vivo***  GE-EPI, 0.22 x 0.2 x 0.4 mm isotropic, 2000 rep. | ***In vivo***  multi-shot SE-EPI, 0.2x0.2x0.4 mm, 90 directions, b-factor=1000-2000 s/mm^2^ | Autism | No / No |

1Refers to the fact that no link to download the data is provided, but that the data are only available upon reasonable request to the authors.

BOLD (blood oxygenation level-dependent), contactin-associated knockout (CNTNAP2^−/−^), d (days), EPI (Echo Planar Imaging), Fragile-X knockout (Fmr1^−/y^), m (months), N/A (not applicable), SWS (rat connectome project in Swanson space), SE-DT-EPI (spin-echo diffusion tensor echo planar imaging), wks (weeks.

**Table 3: Detailed overview of software and algorithms used for MRI data analysis and statistics**

|  | **Study** | **Analysis Programs** | **Analysis Methods** |
| --- | --- | --- | --- |
| 1 | [(Arefin et al., 2017)](https://paperpile.com/c/X6NvQ0/PeFtp) | - MATLAB(The MathWorks, - Natick, MA) along with the fMRI tool of statistical parametric mapping SPM8* and its SPM Mouse toolbox - MATLAB-based toolbox GIFT | **DTI**   - Diffusion-based tractography, HARDI - Global fiber tracking approach   **rs-fMRI & Statistics**   - ICA - Spatially independent components (Infomax algorithm) - Pearson PC analysis (derived with ICASSO algorithm) - Seed-based correlation analysis |
| 2 | [(Asleh et al., 2020)](https://paperpile.com/c/X6NvQ0/6fV2V) | - ExplorDTI: A graphical toolbox for processing, analyzing, and visualizing diffusion MR data - Advanced Normalization Tools (ANTs) | **DTI**   - Robust estimation algorithm - Motion correction, eddy current correction, brain extraction - Normalized FA maps - two-sample Student’s t test   **rs-fMRI**   - Slice time correction, motion correction (rigid-body correction) - Symmetric diffeomorphic image normalization (SyN) - Frame displacement threshold of 50 μm - Motion detection with mean root square method - Thresholding based on 150% the interquartile range above the 75th percentile - Demeaning, detrending - Regressing out mean ventricles and vascular signals - Bandpass filtering (0.009 to 0.08 Hz) - Smoothing using convolution with a Gaussian function (FWHM of 0.6 mm)   **Statistics**   - Seed-to-whole brain correlation map, Fisher’s Z transform, Z(r), map - Seed-to-seed analysis, Pearson’s correlation coefficient, Fisher’s Z transform for cross-subject comparison |
| 3 | [(Degiorgis et al. 2020)](https://paperpile.com/c/X6NvQ0/MVuK) | - MATLAB (The MathWorks, Natick, Massachusetts) - SPM8 with SPMmouse toolbox - SPM deformation function - N4ITK - ANTs - iterative HITS algorihm - Stouffer methods | **DTI**   - **Global fiber tracking** [(Reisert and Kiselev, 2011)](https://paperpile.com/c/X6NvQ0/2oah) - **Fiber density maps**   **fMRI**   - **Partial correlation analysis** - **Seed-based analysis** - **motion correction with realignment** - **Smoothing** - **Parcellation and wrapping** - **Dynamic functional connectivity, using the method described in**   **Statistics**   - **voxel-wise statistical analysis** - **two-sample t-tests** - **partial correlation analysis between each ROI** - **Normalization Fischer’s z-transformation** - **multiple comparisons correction** - **False Discovery Rate correction** |
| 4 | [(Díaz-Parra et al., 2017)](https://paperpile.com/c/X6NvQ0/zDEWA) | - FSLv5.0 (FMRIB Software Library - MATLAB 2014a (The MathWorks,Natick, MA) | **rs-fMRI**   - Voxel scale up by a factor of 10, motion correction, brain segmentation, co-registration - DVARS measure: global intensity normalization (1000) spike detection - Nuisance regression models - Band-pass filtering (nonlinear high-pass filter, Gaussian linear low-pass filter 0.01 to 0.1 Hz) - Smoothing was not applied to avoid introducing spurious high correlations between a node and its neighbors - Co-registering rs-fMRI data with T2w data by a rigid body transformation   **Statistics**   - Outliers: temporal points exceeding the 75th percentile þ 1.5*IQR - Normalized to a rat template (affine deformation) - Z-scoring rs-fmri |
| 5 | [(Grandjean et al., 2017)](https://paperpile.com/c/X6NvQ0/asR0z) | - AFNI_2011_12_21_1014, meica.py script - group-ICA MELODIC - FSL, FMRIB Software Library version 5.0 - R (The R Foundation for Statistical Computing, Vienna, Austria) | **rs-fMRI**   - Motion correction, despiking, skull stripping, ICA - Coregistering using linear affine and nonlinear greedy SyN transformation   **Statistics**   - Seed-based maps - Pearson’s correlation and Fisher’s z transformed, binarized - Individual z-statistics seed-to-target matrices premasked using a t test to consider only connections significantly different from 0 - Partial Spearman’s and Nonparametric Spearman’s correlations - ROC curves, AUC; Permutation testing - Two-way t-test - correction for multiple comparisons by FDR |
| 6 | [(Green et al., 2018)](https://paperpile.com/c/X6NvQ0/Ynryp) | - FMRIB Software Library, FSL SUSAN, customized version of FSLNets v0.6, - DSIStudio - MATLAB v.2014b (The MathWorks, Natick, MA) - GraphPad Prism v. 7.00 (GraphPad Software, La Jolla California USA) | **DTI**   - Brain extraction - Motion correction - Spherical harmonics-based Q-ball reconstruction (rp=0.006, 8-fold tessellation) - Fiber tracking: deterministic streamline whole-brain tractography, voxel-normalization   **rs-fMRI**   - Slice-wise motion correction, linear detrending - Physiological noise removal by regresion - In-plane spatial smoothing with a Gaussian filter of FWHM = 0.3 mm - Bandpass filter of bandwidth 0.01–0.08 Hz - Normalization - Pearson correlation coefficient - Fisher z-transformation   **Statistics**   - Shapiro-Wilk normality test - Friedman test - Kruskal-Wallis analysis of variance - Dunn’s post-hoc correction for multiple comparisons |
| 7 | [(Green et al., 2019)](https://paperpile.com/c/X6NvQ0/YRk9u) | - FSL,FLIRT (FMRIB’s Linear Image Registration Tool, customized version of FSLNets v0.6, - Custom-written scripts - DSIStudio - ImageJ (Version 1.46; National Institutes of Health, Bethesda, MD) - custom macros - Matlab v.2014b (MathWorks) - SPSS 24 software (IBM) | **DTI**   - Brain-extraction - Motion-correction - Coregistering - Spherical harmonics-based Q-ball reconstruction (rp=0.006, 8-fold tessellation) - Quantitative anisotropy maps - Deterministic streamline whole-brain tractography - Voxel-normalization   **rs-fMRI**   - Slice-wise motion-correction, brain-extraction, linear detrending - Smoothing (Gaussian filter of FWHM 0.3 mm) - Bandpass-filtered to 0.01–0.08 Hz, normalizing - Group-wise full Pearson correlation - No global signal removal - Fisher z transformation   **Statistics**   - Univariate repeated-measures ANOVA (cross-correlation, within-group effects) - Mixed ANOVA (cross-correlation between-group effects) - Post-hoc Bonferroni corrections - Friedman test - Dunn post-hoc corrections for multiple comparisons (longitudinal within-group analysis) - Mann–Whitney for pairwise, between-group analysis - Repeated-measures ANOVA for the intranode QA analysis |
| 8 | [(Haberl et al., 2015)](https://paperpile.com/c/X6NvQ0/dv9D2) | - Statistical Parametri Mapping (SPM) mouse toolbox [SPM5, University College London] - ANTs (Advanced Normalization Tools) V1.9 - FEAT tool of FSL [FSL 5.0] - FSLNets (FSLNets, V0.3) - PATCH algorithm^^[[1]](#footnote-1)^^ - Prism 6.0e | **DTI**   - Realignement - Linear affine and nonlinear diffeomorphic transformation   **rs-fMRI**   - Realignment using a least-squares method and rigid-body transformation - linear affine and nonlinear diffeomorphic transformation - Temporal high-pass filtering (cutoff at 0.01Hz) - Movement regression using total correlation analyses - Pearson correlation values were Fisher-transformed to Z scores   **Statistics**   - Two-tailed unpaired t-tests - Mann-Whitney test - Two-sample Kolmogorov-Smirnov test - Holm-Sidak method for Multiple t-tests correction - Bonferroni’s method for multivariate ANOVA correction |
| 9 | [(Hübner et al., 2017)](https://paperpile.com/c/X6NvQ0/KoIop) | - Statistical Parametric - Mapping (SPM8) - SPMmouse for MATLAB (MathWorks) - MATLAB tool GIFT (Group ICA of fMRI Toolbox, v1.3i) - ICASSO - MATLAB In house developed mouse brain atlas tool | **Data pre-processing**   - Realignment - Coregistration - Segmentation - Normalized mutual information approach - 4th degree B-Spline interpolation - 6-parameter rigid body transformation - In-house refined tissue probability maps (TPM) - Gaussian smoothing (0.4×0.4×1 mm³ FWHM)   **DTI**   - Global fiber tracking algorithm - Fiber density (FD) maps - Upscaling and downscaling - Whole brain parcelation - Seed (IC) based structural connectivity assessment   **rs-fMRI**   - Cross-correlation - zero-phase band-pass filter (0.01–0.1 Hz) - Fisher's z-transform - Normalized spatial correlation maps   **Statistics**   - Z-score mapping - Kernighan–Lin algorithm for spectral community detection - Global clustering coefficient (CC) - Average shortest path length, small-worldness - Anderson-Darling test for normal distribution confirmation - Two-sample t-test (Voxel-wise statistical group comparison) - Unpaired two-sample two-sided t-test (structural connectivity differences for specific ROIs) - Identical statistical analysis - Benjamini–Hochberg procedure - Pearson correlation |
| 10 | [(Karatas et al., 2021)](https://paperpile.com/c/X6NvQ0/V3eCI) | - N4ITK - ANTs registration toolbox - SPM12 - in-house-developed DTI and FiberTool software package for SPM | **Data pre-processing**   - Bias field inhomogeneity - Jacobian map - Smoothing with Gaussian kernel (FWHM: 0.2 mm) - General linear model for inter-groups comparisons (Voxel level) - Two-sample t-test - False discovery rate (FDR) correction   **DTI**   - Diffusion based parameter mapping - Diffusion tractography (Global algorithm)   **rs-fMRI**   - least square approach for realignment - Deformable SyN algorithm for registration - Smoothing by Gaussian kernel with FWHM of 0.3 × 0.3 × 0.7 mm3 - Zero-phase band-pass filter (0.01-0.1 Hz) |
| 11 | [(Kesler et al., 2018)](https://paperpile.com/c/X6NvQ0/FSXTY) | - FMRIB Software Library (FSL) View v3.2.0 - Statistical Parametric Mapping v8 - CONN Toolbox v13 - FSL v5.0 - TrackVis v0.6.1 - R statistical package v3.3.2 (“poweRlaw” library) - Network-Based Statistic Toolbox v1.2 - Brain Connectivity Toolbox | **Data pre-processing**   - Manual 3D Brain Mask, realignment, warping, co-registration - Filtering data to the <0.1 Hz range of spontaneous activity - CompCor method to remove motion and physiologic/nonneuronal artifacts - PCA - Cross-correlation - Normalization (Fisher r-to-z transformation)   **DTI**   - Eddy current correction and tensor reconstruction - Deterministic tractography   **rs-fMRI**   - Negative functional edges were zeroed   **Statistics**   - Graph theoretical analysis - AUC , nonparametric permutation testing, two-tailed p-values - Power-law fitting, permutation testing (Family-wise error, false discovery rate) - Multiple linear regression model - False discovery rate, pearson correlation - Path transitivity, column-wise z-scored mean first passage time, neighborhood overlap, and Matching index |
| 12 | [(Mechling et al., 2016)](https://paperpile.com/c/X6NvQ0/xCbUX) | - MATLAB (The MathWorks) - SPM8 (fMRI tool) - MATLAB-based toolbox GIFT - FiberTool package developed in-house | **Data pre-processing**   - Registration, alignment (linear and nonlinear registration), B-Spline deformation, normalization - Smoothing (Gaussian kernel of FWHM 0.4 × 0.4 × 1)   **DTI**   - Fisher’s z transformation - Global mouse brain fiber tractography, HARDI   **rs-fMRI**   - High-dimensional ICA (100 components) - Spatial group ICA - Color-coded z-maps - Coregistration with AMBA - Partial Pearson Correlation - Fisher’s z transformation - Two-sided one-sample t test |
| 13 | [(van Meer et al., 2010)](https://paperpile.com/c/X6NvQ0/kIUdG) |  | **rs-fMRI**   - Motion correction, spatial smoothing, linear regression - Band-pass filter with 0.01 < f < 0.1 Hz - Fisher-transformed correlation coefficient   **PW-MRI**   - Group mean ΔR1 maps (MnCl2 injection)   **Statistics**   - Two-way repeated measures ANOVA (analysis of variance) - Post hoc Bonferroni’s testing |
| 14 | [(van Meer et al., 2012)](https://paperpile.com/c/X6NvQ0/ikjNG) | - Elastix - FMRIB, FSL - AFNI - R, stats package for the Mann–Whitney U and Spearman’s rank correlation tests - R, nlme and lme4 packages for the linear mixed model analyses | **rs-fMRI**   - Nonrigid image registration, affine-only registration - Spatial smoothing with an isotropic Gaussian kernel of 1.0mmfull-width at half-maximum - Rigid-body motion correction - Bandpass filter at 0.01<f< 0.1 Hz   **DTI**   - Multivariate fitting and diagonalization: FA maps   **Statistics**   - Mann–Whitney U test - Repeated-measures linear - Mixed model analysis with fixed effects “group”, “time” and “group time,” and random effect “subject” - Post-hoc Tukey’s testing - Linear mixed model analysis - Probabilistic independent component analysis - Thresholding at Z=4 - Fisher-transformed correlation coefficient - Tract-based spatial statistics - Nonparametric, permutation-based t-testing |
| 15 | [(Melozzi et al., 2019)](https://paperpile.com/c/X6NvQ0/Qcny4) | - Virtual Brain software (reduced Wong Wang model) - The Virtual Mouse Brain (TVMB) - MRtrix3 software (iFOD2 and SD_Stream) | **DTI**   - Fiber directionality   **rs-fMRI**   - Simulating resting state dynamics (reduced WongWang model) - Balloon-Windekessel method   **Statistics**   - Welch’s test - Bonferroni correction - Global signal regression - Normalized U statistics of the Mann–Whitney U |
| 16 | [(Muñoz-Moreno et al., 2018)](https://paperpile.com/c/X6NvQ0/zmTU3) | - ANTs - N4ITK algorithm - SPM8 software - FSL - NiTime | **Data pre-processing**   - Skull stripping, tissue segmentation, parcellation, tissue probability maps (TPMs) - Nonlocal means denoising filter   **DTI**   - Eddy current correction, bias correction - Spin EPI distortion correction   **rs-fMRI**   - Slice time correction, motion correction - Z-score normalization - Detrending - Smoothing with an FWHM of 1.2 mm - Frequency filtering (0.01-0.1 Hz)   **Statistics**   - Kruskal-Wallis tests (statistical significance between groups) - Generalized linear modeling - Multiple comparisons correction (false discovery rate -FDR) - Spearman’s correlation coefficient - Mini Mental State Examination scores |
| 17 | [(Muñoz-Moreno et al., 2020)](https://paperpile.com/c/X6NvQ0/mNt8B) | - Dipy - SPM8 - ANTs - NiTime - network-based statistics (NBS) toolbox | **DTI & rs-fMRI**   - Same as 2018 - Deterministic tractography based on constrained spherical deconvolution model - Z-score normalization - Detrending, smoothing with an FWHM of 1.2 mm - Frequency filtering (0.01-0.1 Hz)   **Statistics**   - Linear mixed-effects Linear mixed-effects models: (LME) models - Multiple comparisons correction (false discovery rate -FDR) - Spearman’s correlation coefficient - Kruskal-Wallis tests |
| 18 | [(Parent et al., 2020)](https://paperpile.com/c/X6NvQ0/T4ISq) | - BioImage Suite software - AFNI - 3dTshift - 3dvolreg | **Data pre-processing**   - Linear registration, tensor model fitting   **rs-fMRI**   - Slice time correction - Motion correction - Spatial smoothing using a Gaussian filter (full width at half maximum = 1.5 mm) - Linear detrending - Band-pass filtered (0.01–0.15 Hz) - Fisher’s z-transformation   **DTI**   - Band-pass filtered (0.01–0.15 Hz) - Fisher’s z-transformation |
| 19 | [(Schroeter et al., 2017)](https://paperpile.com/c/X6NvQ0/aOp2I) | - AFNI - MATLAB-based software Aedes - Custom MATLAB code - FSL - R (3.0.1) | **Data pre-processing**   - Co-registration   **rs-fMRI**   - Slice-time correction, motion correction - Weak Gaussian blur (FWHM=0.3 mm) - Bandpass-filtered for 0.01 Hz to 0.3 Hz - Global signal regression - Detrending by adding polynomials   **DTI**   - Eddy current correction - Likelihood test ratio   **Statistics**   - One sample t-test - Pearson’s correlations - Fisher’s z correction - False discovery rate correction |
| 20 | [(Sethi et al., 2017)](https://paperpile.com/c/X6NvQ0/gY8nt) | - MELODIC - MCFLIRT - FSL-FIX - ANTs v2.1 - Timeseries analysis software package hctsa (v0.91, github.com/benfulcher/hctsa) | **Data pre-processing**   - Co-registration - Skull-stripping   **DTI**   - AMBMC template   **rs-fMRI**   - Removal of unwanted confounds from the time series - Within-subject spatial-ICA, high-pass filtering (>0.01 Hz), correction for head motion - In-plane smoothing with a 0.3x0.3 mm kernel - Removal of the variance of the artifactual component   **Statistics**   - Holm–Bonferroni method, Mass univariate testing with family-wise error correction - Partial Spearman correlation coefficient, Autocorrelation - Gaussian estimator at time lag, Measures of randomness, Ljung-Box Q-test - Normalized permutation entropy - Power spectral analysis, Right-tailed partial Spearman correlations |
| 21 | [(Straathof et al., 2020)](https://paperpile.com/c/X6NvQ0/ihMYD) | - FMRIB’s Software Library (FSL) v5.0 - FNIRT - MCFLIRT - BET - iFOD2 algorithm MRtrix3 - R (version 3.2.3) | **Data pre-processing**   - Linear & non-linear registration - Motion-correction, brain Extraction - Paxinos and Watson rat brain atlas   **DTI (Post-mortem)**   - Constrained spherical deconvolution tractography - Filtering with SIFT   **rs-fMRI**   - No global signal regression - Band-pass filtering between 0.01 and 0.1 Hz - ICA (20 components) - Fisher’s Z-transformed full correlation coefficients   **Statistics**   - Two-tailed Spearman rank correlation coefficient - Spearman rank correlation coefficients - Bootstrapping - Average Euclidean length |
| 22 | [(Vega-Pons et al., 2016)](https://paperpile.com/c/X6NvQ0/kRcQO) | - - | **DTI**   - Projections with a deterministic fiber assignment using the continuous tracking algorithm - FACT deterministic algorithm for white matter tractography - Anisotropy threshold (values below 0.15), Maximum stiffness condition   **rs-fMRI**   - Co-registration, Motion correction, - Band-pass filtered to frequency window of 0.01–0.08Hz, Spatial smoothing (FWHM: 0.6 mm)   **Statistics**   - Variance normalization - Pairwise pearson correlation coefficients - Kernel Two-Sample Test (KTST) - Support Vector Machines (SVM) |
| 23 | [(Zerbi et al., 2018)](https://paperpile.com/c/X6NvQ0/Yd1y4) | - ANTs v2.1 - NDP.view2 - FSLNets | **rs-fMRI**   - Removal of unwanted confounds, artifact removal, despiking, band-pass filtered (0.01–0.3 Hz) - Co-registration, Skull stripping, Normalization - AMBMC templates   **DTI**   - Realignment, Eddy current correction, Tensor estimation, Normalization   **Fluorescence Microscopy**   - Mosaic wide-field fluorescence acquisition system   **Statistics**   - Regularized Pearson’s correlation coefficients |

References

[Arefin, T. M., Mechling, A. E., Meirsman, A. C., Bienert, T., Hübner, N. S., Lee, H.-L., et al. (2017). Remodeling of Sensorimotor Brain Connectivity in Gpr88-Deficient Mice. *Brain Connect.* 7, 526–540.](http://paperpile.com/b/X6NvQ0/PeFtp)

[Asleh, J., Shofty, B., Cohen, N., Kavushansky, A., López-Juárez, A., Constantini, S., et al. (2020). Brain-wide structural and functional disruption in mice with oligodendrocyte-specific deletion is rescued by inhibition of nitric oxide synthase. *Proc. Natl. Acad. Sci. U. S. A.* 117, 22506–22513.](http://paperpile.com/b/X6NvQ0/6fV2V)

[Degiorgis, L., Karatas, M., Sourty, M., Faivre, E., Lamy, J., Noblet, V., et al. (2020). Brain network remodelling reflects tau-related pathology prior to memory deficits in Thy-Tau22 mice. *Brain* 143, 3748–3762.](http://paperpile.com/b/X6NvQ0/MVuK)

[Díaz-Parra, A., Osborn, Z., Canals, S., Moratal, D., and Sporns, O. (2017). Structural and functional, empirical and modeled connectivity in the cerebral cortex of the rat. *Neuroimage* 159, 170–184.](http://paperpile.com/b/X6NvQ0/zDEWA)

[Grandjean, J., Zerbi, V., Balsters, J. H., Wenderoth, N., and Rudin, M. (2017). Structural Basis of Large-Scale Functional Connectivity in the Mouse. *J. Neurosci.* 37, 8092–8101.](http://paperpile.com/b/X6NvQ0/asR0z)

[Green, C., Minassian, A., Vogel, S., Diedenhofen, M., Beyrau, A., Wiedermann, D., et al. (2018). Sensorimotor Functional and Structural Networks after Intracerebral Stem Cell Grafts in the Ischemic Mouse Brain. *J. Neurosci.* 38, 1648–1661.](http://paperpile.com/b/X6NvQ0/Ynryp)

[Green, C., Sydow, A., Vogel, S., Anglada-Huguet, M., Wiedermann, D., Mandelkow, E., et al. (2019). Functional networks are impaired by elevated tau-protein but reversible in a regulatable Alzheimer’s disease mouse model. *Mol. Neurodegener.* 14, 13.](http://paperpile.com/b/X6NvQ0/YRk9u)

[Haberl, M. G., Zerbi, V., Veltien, A., Ginger, M., Heerschap, A., and Frick, A. (2015). Structural-functional connectivity deficits of neocortical circuits in the Fmr1 (-/y) mouse model of autism. *Sci Adv* 1, e1500775.](http://paperpile.com/b/X6NvQ0/dv9D2)

[Hübner, N. S., Mechling, A. E., Lee, H.-L., Reisert, M., Bienert, T., Hennig, J., et al. (2017). The connectomics of brain demyelination: Functional and structural patterns in the cuprizone mouse model. *Neuroimage* 146, 1–18.](http://paperpile.com/b/X6NvQ0/KoIop)

[Karatas, M., Noblet, V., Nasseef, M. T., Bienert, T., Reisert, M., Hennig, J., et al. (2021). Mapping the living mouse brain neural architecture: strain-specific patterns of brain structural and functional connectivity. *Brain Struct. Funct.* 226, 647–669.](http://paperpile.com/b/X6NvQ0/V3eCI)

[Kesler, S. R., Acton, P., Rao, V., and Ray, W. J. (2018). Functional and structural connectome properties in the 5XFAD transgenic mouse model of Alzheimer’s disease. *Netw Neurosci* 2, 241–258.](http://paperpile.com/b/X6NvQ0/FSXTY)

[Mechling, A. E., Arefin, T., Lee, H.-L., Bienert, T., Reisert, M., Ben Hamida, S., et al. (2016). Deletion of the mu opioid receptor gene in mice reshapes the reward-aversion connectome. *Proc. Natl. Acad. Sci. U. S. A.* 113, 11603–11608.](http://paperpile.com/b/X6NvQ0/xCbUX)

[Melozzi, F., Bergmann, E., Harris, J. A., Kahn, I., Jirsa, V., and Bernard, C. (2019). Individual structural features constrain the mouse functional connectome. *Proc. Natl. Acad. Sci. U. S. A.* 116, 26961–26969.](http://paperpile.com/b/X6NvQ0/Qcny4)

[Muñoz-Moreno, E., Tudela, R., López-Gil, X., and Soria, G. (2018). Early brain connectivity alterations and cognitive impairment in a rat model of Alzheimer’s disease. *Alzheimers. Res. Ther.* 10, 16.](http://paperpile.com/b/X6NvQ0/zmTU3)

[Muñoz-Moreno, E., Tudela, R., López-Gil, X., and Soria, G. (2020). Brain connectivity during Alzheimer’s disease progression and its cognitive impact in a transgenic rat model. *Netw Neurosci* 4, 397–415.](http://paperpile.com/b/X6NvQ0/mNt8B)

[Parent, M., Chitturi, J., Santhakumar, V., Hyder, F., Sanganahalli, B. G., and Kannurpatti, S. S. (2020). Kaempferol treatment after traumatic brain injury during early development mitigates brain parenchymal microstructure and neural functional connectivity deterioration at adolescence. *J. Neurotrauma* 37, 966–974.](http://paperpile.com/b/X6NvQ0/T4ISq)

[Reisert, M., and Kiselev, V. G. (2011). Fiber continuity: an anisotropic prior for ODF estimation. *IEEE Trans. Med. Imaging* 30, 1274–1283.](http://paperpile.com/b/X6NvQ0/2oah)

[Schroeter, A., Grandjean, J., Schlegel, F., Saab, B. J., and Rudin, M. (2017). Contributions of structural connectivity and cerebrovascular parameters to functional magnetic resonance imaging signals in mice at rest and during sensory paw stimulation. *J. Cereb. Blood Flow Metab.* 37, 2368–2382.](http://paperpile.com/b/X6NvQ0/aOp2I)

[Sethi, S. S., Zerbi, V., Wenderoth, N., Fornito, A., and Fulcher, B. D. (2017). Structural connectome topology relates to regional BOLD signal dynamics in the mouse brain. *Chaos* 27, 047405.](http://paperpile.com/b/X6NvQ0/gY8nt)

[Straathof, M., Sinke, M. R. T., Roelofs, T. J. M., Blezer, E. L. A., Sarabdjitsingh, R. A., van der Toorn, A., et al. (2020). Distinct structure-function relationships across cortical regions and connectivity scales in the rat brain. *Sci. Rep.* 10, 56.](http://paperpile.com/b/X6NvQ0/ihMYD)

[van Meer, M. P. A., Otte, W. M., van der Marel, K., Nijboer, C. H., Kavelaars, A., van der Sprenkel, J. W. B., et al. (2012). Extent of bilateral neuronal network reorganization and functional recovery in relation to stroke severity. *J. Neurosci.* 32, 4495–4507.](http://paperpile.com/b/X6NvQ0/ikjNG)

[van Meer, M. P. A., van der Marel, K., Otte, W. M., Berkelbach van der Sprenkel, J. W., and Dijkhuizen, R. M. (2010). Correspondence between altered functional and structural connectivity in the contralesional sensorimotor cortex after unilateral stroke in rats: a combined resting-state functional MRI and manganese-enhanced MRI study. *J. Cereb. Blood Flow Metab.* 30, 1707–1711.](http://paperpile.com/b/X6NvQ0/kIUdG)

[Vega-Pons, S., Olivetti, E., Avesani, P., Dodero, L., Gozzi, A., and Bifone, A. (2016). Differential Effects of Brain Disorders on Structural and Functional Connectivity. *Front. Neurosci.* 10, 605.](http://paperpile.com/b/X6NvQ0/kRcQO)

[Zerbi, V., Ielacqua, G. D., Markicevic, M., Haberl, M. G., Ellisman, M. H., A-Bhaskaran, A., et al. (2018). Dysfunctional autism risk genes cause circuit-specific connectivity deficits with distinct developmental trajectories. *Cereb. Cortex* 28, 2495–2506.](http://paperpile.com/b/X6NvQ0/Yd1y4)

1. M. P. Zwiers, Patching cardiac and head motion artefacts in diffusion-weighted images.

   Neuroimage 53, 565–575 (2010 [↑](#footnote-ref-1)
